# Trans-anal small bowel evisceration through a perforation of the rectum: a case report

**DOI:** 10.1093/jscr/rjag600

**Published:** 2026-07-16

**Authors:** Elynn Ong, Apoorva Saboo, Damien Loh, Ee Jun Ban, Peter Carne

**Affiliations:** Department of Surgery, Alfred Health, Melbourne, VIC, Australia; Department of Surgery, Alfred Health, Melbourne, VIC, Australia; Department of Surgery, Alfred Health, Melbourne, VIC, Australia; Department of Surgery, Alfred Health, Melbourne, VIC, Australia; Department of Surgery, Alfred Health, Melbourne, VIC, Australia

**Keywords:** case reports, trans-anal small bowel evisceration, rectal perforation, intestinal perforation

## Abstract

Trans-anal small bowel evisceration (TSBE) is a rare, life-threatening surgical emergency, typically presenting with a patient who is unwell. We report an atypical case of a stable 78-year-old female with a history of chronic constipation presenting with a reducible TSBE. Exploratory laparotomy demonstrated an anterior rectal defect. The defect was repaired primarily, and small bowel resection was performed. This case highlights the diagnostic difficulties that clinicians may face due to the rarity of this condition, particularly in a well-appearing patient, and underscores the importance of prompt definitive surgical management.

## Introduction

Trans-anal intestinal herniation was first reported in 1827 [1]. Since then, few cases have been described in literature and predominantly relate to two main risk factors; chronic rectal prolapse and/or rectal trauma. The earliest reported case was published in 1984 with trans-anal evisceration of the entire length of non-viable small bowel highlighting the dire consequences of this rare condition [2]. The proposed pathophysiology involves small bowel loops forming a sac within the pouch of Douglas and exerting pressure on a weakened rectal wall, often in the context of chronic prolapse. Trauma-related cases are usually iatrogenic or secondary to penetrating or blunt injury [3, 4]. The clinical presentation of trans-anal small bowel evisceration (TSBE) is usually dramatic with irreducible, congested loops of small bowel with peritonitis. We present an atypical case of a clinically well patient with TSBE and its associated diagnostic challenges.

## Case report

Informed consent for publication of the research details and clinical images was obtained from the patient. All study procedures were conducted in accordance with the principles of the Declaration of Helsinki.

A 78-year-old woman presented to the Emergency Department after developing a large, painful rectal prolapse during straining, which she subsequently reduced herself. She reported no prior episodes of recurrent prolapse. Her medical history included chronic constipation, osteoarthritis, and dyslipidaemia. She denied recent rectal instrumentation, trauma, previous abdominal or pelvic surgery, or gynaecological procedures.

**Figure 1:**
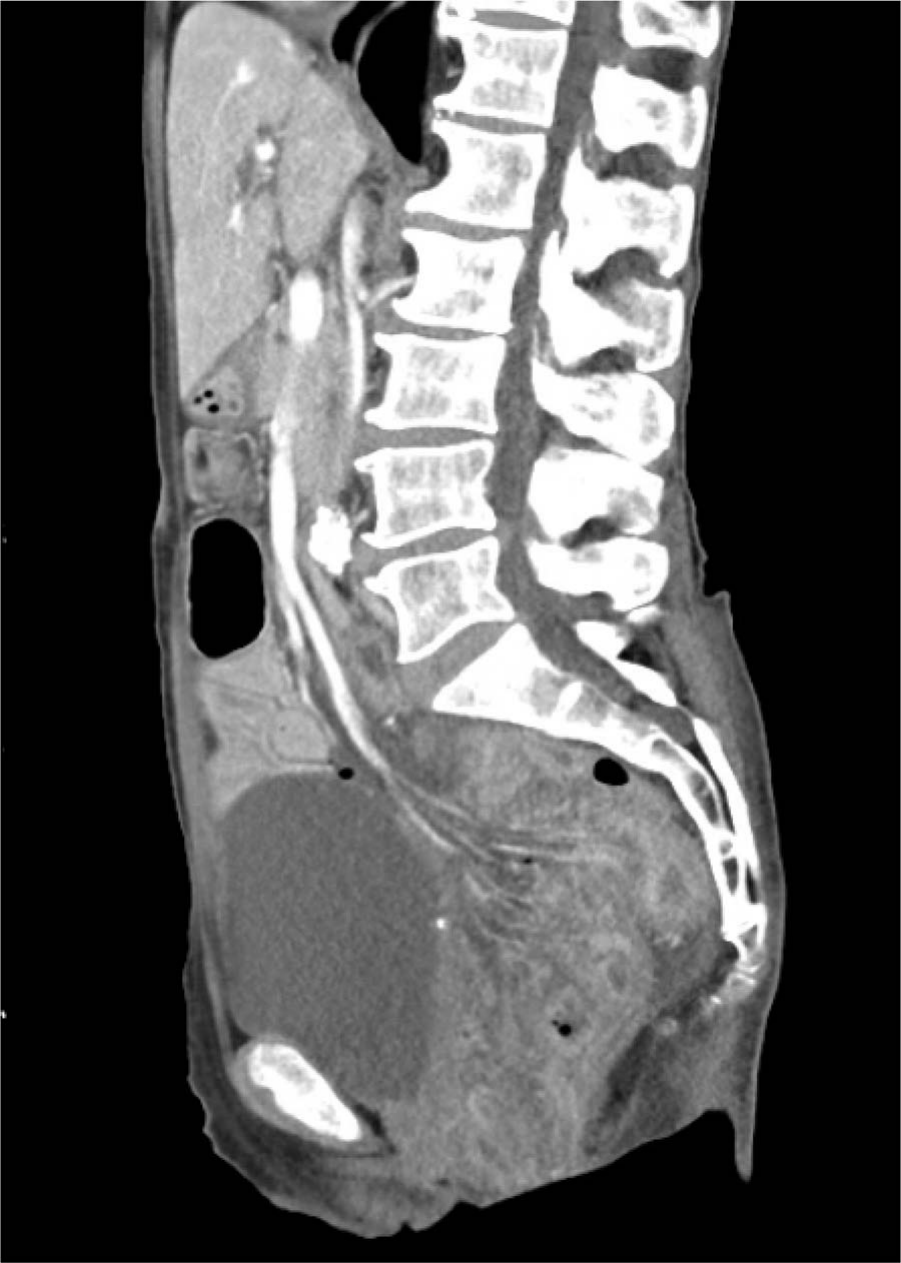
Sagittal view of contrast enhanced CT showing small bowel prolapse into the pelvis.

**Figure 2:**
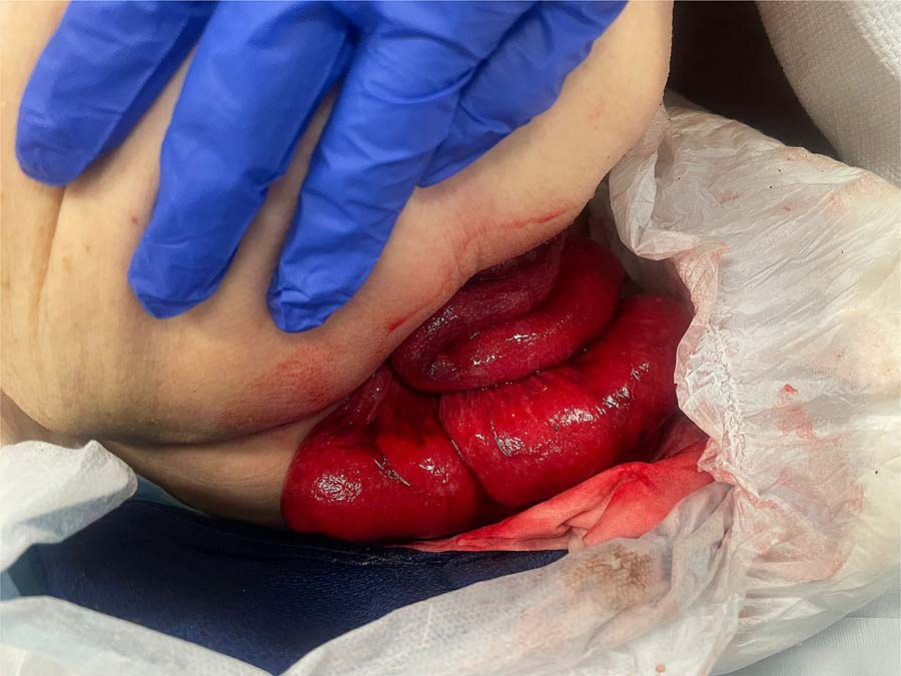
Transanal small bowel evisceration.

On examination, there was no visible prolapse, and no recurrence was elicited with straining or standing. Abdominal examination revealed a soft, non-distended abdomen without tenderness, peritonism, or features of obstruction. Digital rectal examination did not demonstrate any obvious mucosal defect or mass. Contrast-enhanced computed tomography (CT) of the abdomen and pelvis demonstrated appearances consistent with possible rectal prolapse with small bowel loops in the recto-uterine pouch, but there was no free gas, free fluid, or pneumoperitoneum (Fig. 1). The patient remained haemodynamically stable and was admitted for observation. During admission, she developed recurrent prolapse with ~30 cm of small bowel and mesentery eviscerating trans-anally (Fig. 2).

**Figure 3:**
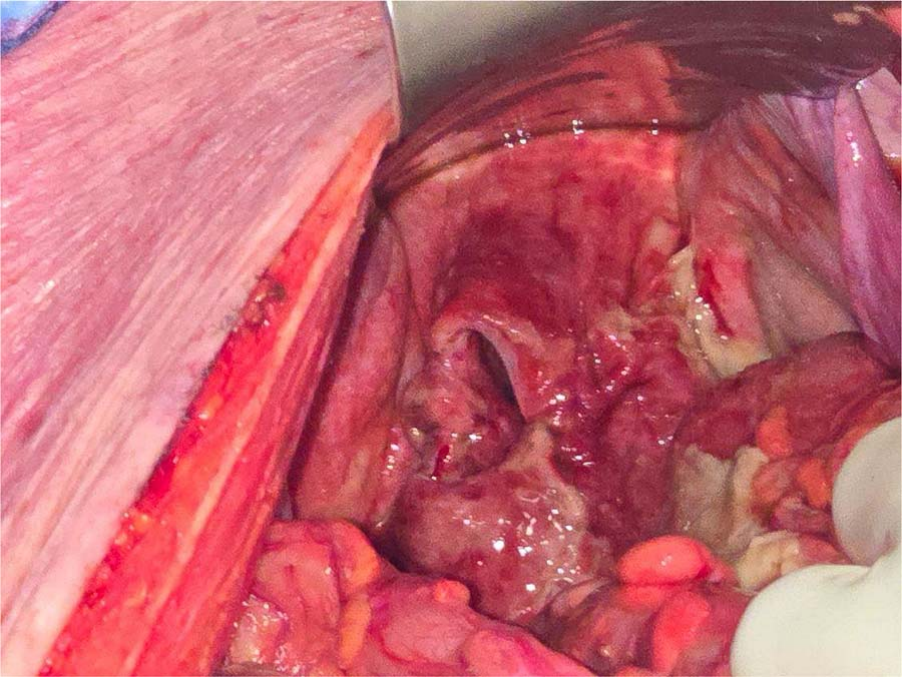
Anterior rectal defect.

**Figure 4:**
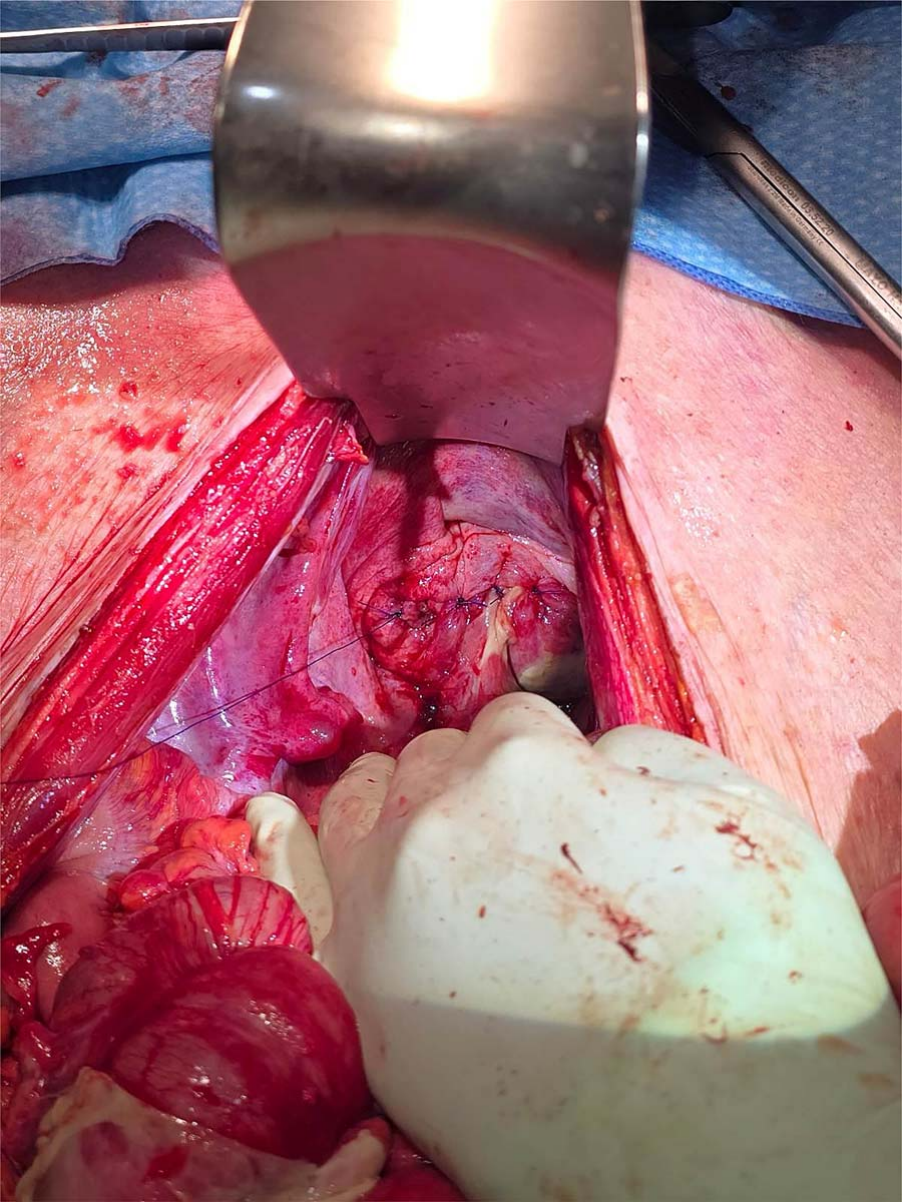
Repaired rectal defect.

She was taken urgently to theatre for examination under anaesthesia and exploratory laparotomy. Intraoperatively, a 3 × 3 cm anterior rectal wall defect was identified ~8 cm above the anal verge and peritoneal reflection (Fig. 3). The defect had clean, punched-out margins without macroscopic evidence of malignancy or necrosis. Biopsies were obtained. The defect was closed primarily using absorbable polydioxanone sutures in a double-layer fashion (Fig. 4). Intraoperative sigmoidoscopy confirmed luminal patency and a negative air leak test. A diverting loop colostomy was fashioned proximally. The eviscerated small bowel appeared oedematous with multiple serosal tears. A 20-cm segment was resected, and a stapled anastomosis was performed.

The patient’s postoperative course was uneventful. Histopathology demonstrated ulceration at the rectal defect without evidence of malignancy.

## Discussion

This case emphasizes the broad differential diagnosis for a prolapsing rectal mass; of which rectal prolapse is only one entity. Other important differentials include haemorrhoids, polyps, malignancy, rectal intussusception and, more rarely, trans-anal organ evisceration. As management differs substantially between benign and malignant pathology and between full-thickness and mucosal prolapse, accurate characterization of the underlying pathology is crucial.

Our case underscores the central importance of a focused history and meticulous physical examination. The absence of a typical history of recurrent rectal prolapse, together with preceding traumatic manual disimpaction and straining, should prompt consideration of alternative aetiologies, including stercoral ulceration and subsequent perforation. Furthermore, the spontaneous reduction of the presumed ‘prolapse’ prior to assessment and its failure to recur with straining are atypical for a conventional full-thickness rectal prolapse and should raise diagnostic suspicion [5]. The eventual identification of mesentery within the prolapsing mass is a key discriminating feature, strongly suggestive of TSBE rather than simple mucosal or full-thickness prolapse. This highlights the need to maintain a broad differential diagnosis, even when dealing with seemingly straightforward presentations.

TSBE remains extremely rare, with most reported cases associated with predisposing factors such as rectal prolapse, chronic constipation, diverticular disease or malignancy, and typically triggered by a sudden increase in intra-abdominal pressure (e.g. straining or coughing) [6]. In this patient, a presumed stercoral ulcer likely perforated during straining, facilitating small bowel herniation through the rectal wall.

Gastrointestinal perforation usually presents with sepsis and generalized peritonitis due to faecal contamination of the peritoneal cavity [7]. However, in selected cases, including the present one, systemic signs may be minimal. One proposed explanation is that the herniated bowel loops function as a plug, limiting intraperitoneal contamination such as in our patient. CT is a valuable adjunct in the evaluation of these patients but is not definitive [8]. In particular, the absence of free air or fluid does not exclude TSBE. In this case, the initial CT did not demonstrate pneumoperitoneum, underscoring the primacy of clinical judgement over imaging alone.

Trans-anal small bowel evisceration is a rare but critical diagnosis that can mimic uncomplicated rectal prolapse, especially in hemodynamically stable patients without overt peritonitis. This case reinforces that meticulous history-taking and thorough clinical examination is paramount in patients presenting with a prolapsing rectal mass, particularly when atypical features are present. While cross-sectional imaging can assist in evaluation, it should not delay operative management in the context of high clinical suspicion. Prompt surgical intervention with repair of the rectal defect, assessment, and resection of compromised bowel, and consideration of faecal diversion is essential to minimize morbidity and mortality. Clinicians should remain vigilant for this rare entity, even in well-appearing patients, to avoid potentially catastrophic delays in treatment.
